# The Antidepressant *Trans*-2-Phenylcyclopropylamine Protects Mice from High-Fat-Diet-Induced Obesity

**DOI:** 10.1371/journal.pone.0089199

**Published:** 2014-02-21

**Authors:** Adi Shemesh, Arian Abdulla, Fajun Yang, Streamson C. Chua, Jeffrey E. Pessin, Haihong Zong

**Affiliations:** 1 Department of Molecular Pharmacology, Albert Einstein College of Medicine, Bronx, New York, United States of America; 2 Department of Developmental and Molecular Biology, Albert Einstein College of Medicine, Bronx, New York, United States of America; 3 Department of Medicine, Albert Einstein College of Medicine, Bronx, New York, United States of America; Sapienza University of Rome, Italy

## Abstract

Mice treated with the antidepressant *trans*-2-phenylcyclopropylamine (2-PCPA) were protected against diet-induced-obesity, and adiposity was reversed in pre-established diet-induced obese mice. Contrary to a recent report that inhibition of lysine-specific demethylase-1 by 2-PCPA results in increased energy expenditure, long-term 2-PCPA treatment had no such effect but its protection against obesity was associated with increased spontaneous locomotor activity, Moreover, pair feeding to assure equal caloric intake in wild type mice as well as in genetic hyperphagic mice (ob/ob) also resulted in weight reduction in 2-PCPA treated mice that correlated with increased activity but no change in energy expenditure. Similarly, short-term intraperitoneal injections of 2-PCPA did not affect food intake but caused a substantial increase in locomotor activity in the light cycle that correlated with increased energy expenditure, whereas activity and energy expenditure were unchanged in the dark cycle. Lastly, 2-PCPA was also effective in reducing obesity in genetic UCP1 null mice. These data suggest that 2-PCPA can reduce obesity by decreasing food intake in the long term while increasing activity in the short-term. However, the protective and weight loss effects of 2-PCPA are independent of UCP1-regulated thermogenesis or basal energy expenditure.

## Introduction

The worldwide obesity epidemic is continuing to increase and has doubled since 1980 with an estimated 500 million obese adults and 40 million children under the age of five being overweight. As obesity is the major predisposing factor for the development of type 2 diabetes, cardiovascular, and non-alcoholic fatty liver diseases with associated morbidity and mortality, varied approaches have been proposed for preventing or reversing the obesity burden.

Early studies on antidepressants have reported weight loss as a side effect of certain drug classes. In particular, the antidepressant *trans*-2-phenylcyclopropylamine (2-PCPA) was associated with weight loss in rat and mouse models of diet- and genetic-induced obesity [1]. 2-PCPA was originally identified as an irreversible nonselective monoamine oxidase inhibitor that exerts its psychotropic actions by increasing levels of central nervous system amine neurotransmitters [2]. Although weight loss was originally attributed to the changes in neurotransmitters as well, recent studies have also found that 2-PCPA is a suicide inhibitor of the flavin adenosine dinucleotide (FAD)-dependent lysine-specific-demethylase 1, LSD1 [3]. Moreover, knockdown or 2-PCPA inhibition of LSD1 reduced adipogenesis, increased the expression of energy expenditure genes associated with mitochondrial metabolism in cultured 3T3-L1 adipocytes and in adipose tissue of high-fat-diet-fed mice, and increased oxygen consumption in 3T3-L1 adipocytes [4].

In this study we demonstrate that long-term 2-PCPA treatment results in a marked resistance to diet-induced-obesity. Whereas energy expenditure was unchanged, food intake was reduced. Food intake reduction appeared to be an adaptation to 2-PCPA-induced weight loss because short-term treatment of wild type (WT) and uncoupling protein 1 (UCP1) knockout mice reduced obesity without affecting food intake. Moreover, core body temperature and energy expenditure of WT mice increased during the light cycle, and obesity and core body temperature alterations were independent of changes in UCP1 and occurred in UCP1 knockout mice. Thus in contrast to previous reports, we conclude that orally administered 2-PCPA treatment reduces adiposity primarily by increasing spontaneous locomotor activity without significantly affecting energy expenditure or food intake.

## Experimental Procedures

### Ethics Statement

This study was carried out in strict accordance with the recommendations in the Guide for the Care and Use of Laboratory Animals of the National Institutes of Health. The protocol was approved by the Albert Einstein College of Medicine Institutional Animal Care and Use Committee (Permit Number: A3312-01). All surgery was performed under sodium pentobarbital anesthesia, and all efforts were made to minimize suffering.

### Animal Models and Experimental Design

C57BL/6J and B6.129-Ucp1tm1Kz/J (UCP1 knockout) and ob/ob mice were purchased from Jackson Laboratory (Bar Harbor, ME) and housed in individual cages at an ambient temperature of 22°C. All male mice at eight weeks of age were acclimated for a week prior to experimentation. Groups of mice were fed either a normal chow diet (NCD) containing 10% calories from fat or a high fat diet (HFD) containing 60% calories from fat (OpenSource Diets, New Brunswick, NJ) for the durations indicated in figure legends.

For the parenteral short-term experiment, 10 mg 2-PCPA/kg body weight or normal saline (control) was injected intraperitoneally once every other day, as previously described [4], for a total of three injections. For enteral short- and long-term experiments, 500 mg of 2-PCPA were mixed into 1 kg of moist powder NCD as previously described [1] and food pellets were formed and air-dried overnight. Due to the reduced amount of HFD intake, 850 mg of 2-PCPA were mixed with 1 kg of HFD to provide an equal dose per lean mass. In the caloric restriction experiment, mice were fed a HFD with or without 2-PCPA ad libitum for 21 days. On the 22nd day, each mouse was allotted 70% of its food intake for 17 additional days. For pair fed, 8 weeks ob/ob age-matched mice were signed to PCPA treated experimental group and control group with NCD. The amounts of food consumed were adjusted by PCPA-treated experimental group on the previous day for 2 months. Also C57BL/6J mice were place on HFD with/without PCPA treated. The amounts of food consumed were controlled by PCPA treated experimental group on the previous day for 2 months.

### Intraperitoneal Glucose and Insulin Tolerance Tests

Glucose tolerance was determined following a 12-hour fast by an intraperitoneal injection of d-glucose (1 g/kg lean mass). Blood samples were drawn at 0, 15, 30, 60, and 120 minutes after injection for measuring plasma glucose using a glucose monitor (Precision Xtra, Bedford, MA) and insulin using an ultrasensitive mouse ELISA insulin kit (Crystal Chem, Downers Grove, IL). For the insulin tolerance test, mice were fasted for 4 hours and injected with insulin (1 U/kg lean mass). Plasma glucose was measured at 0, 15, 30, 60, 120, and 180 minutes following injection.

### Body Mass and Composition

Mice were weighed before and after starting 2-PCPA administration. Fat and lean masses were quantified using EchoMRI-3in1 (Echo Medical Systems, Houston, TX). In addition, fat tissue of long-term HFD-fed mice was weighed immediately following excision.

### Histological Analysis of Adipose Tissue

Inguinal, epididymal, and omental white adipose tissues and interscapular brown adipose tissue were harvested following sacrifice. Formaldehyde-fixed paraffin-embedded fragments of Inguinal, omental, and interscapular adipose tissues were sectioned (5 µm thick) and subjected to standard hematoxylin and eosin staining.

### Indirect Calorimetry

Mice were housed individually in Oxymax Lab Animal Monitoring System cages (Columbus Instruments, Columbus, OH) at an ambient temperature of 22°C and acclimated for one day. Experiments started the following day at the beginning of the 12-hour light cycle and lasted for two days. Mice were fasted during the light cycle and fed ad libitum their respective diets during the dark cycle. The data were normalized by lean mass.

### Core Body Temperature

Core body temperature in conscious mice was recorded on a Physitemp BAT-10 digital thermometer after gentle insertion of a flexible rectal probe (Physitemp, Clifton, NJ).

### Immunoblotting

Adipose tissue was homogenized, and tissue extracts were subjected to reducing SDS-PAGE and blotted using standard procedures. The antibody for UCP1 was purchased from Abcam (Cambridge, MA), for the voltage-dependent anion channel (VDAC) from Cell Signaling (Danvers, MA), and for glyceraldehyde 3-phosphate dehydrogenase (GAPDH) from MBL International Corporation (Woburn, MA). The secondary antibodies were near-infrared fluorescent dye conjugates IRDye 680RD and IRDye 800CW purchased from LI-COR Biosciences (Lincoln, NE).

### Statistical Analysis

Results are presented as mean values ± standard error of the mean (SEM). The difference between means was analyzed using two-sided student’s t-test and was set to be significant if p<0.05. For multiple comparisons, the data were analyzed by two-way analysis of variance (ANOVA) followed by the Student-Newman-Keuls multiple range test. Statistical analyses were made at a significance level of p<0.05 using Graphpad software Prism 6 version (Graphpad software, Inc.). Identical letters indicate bars that are not statistically significant from each other.

## Results

### 2-PCPA Protects Against High Fat Diet-induced Obesity

**Figure 1:**
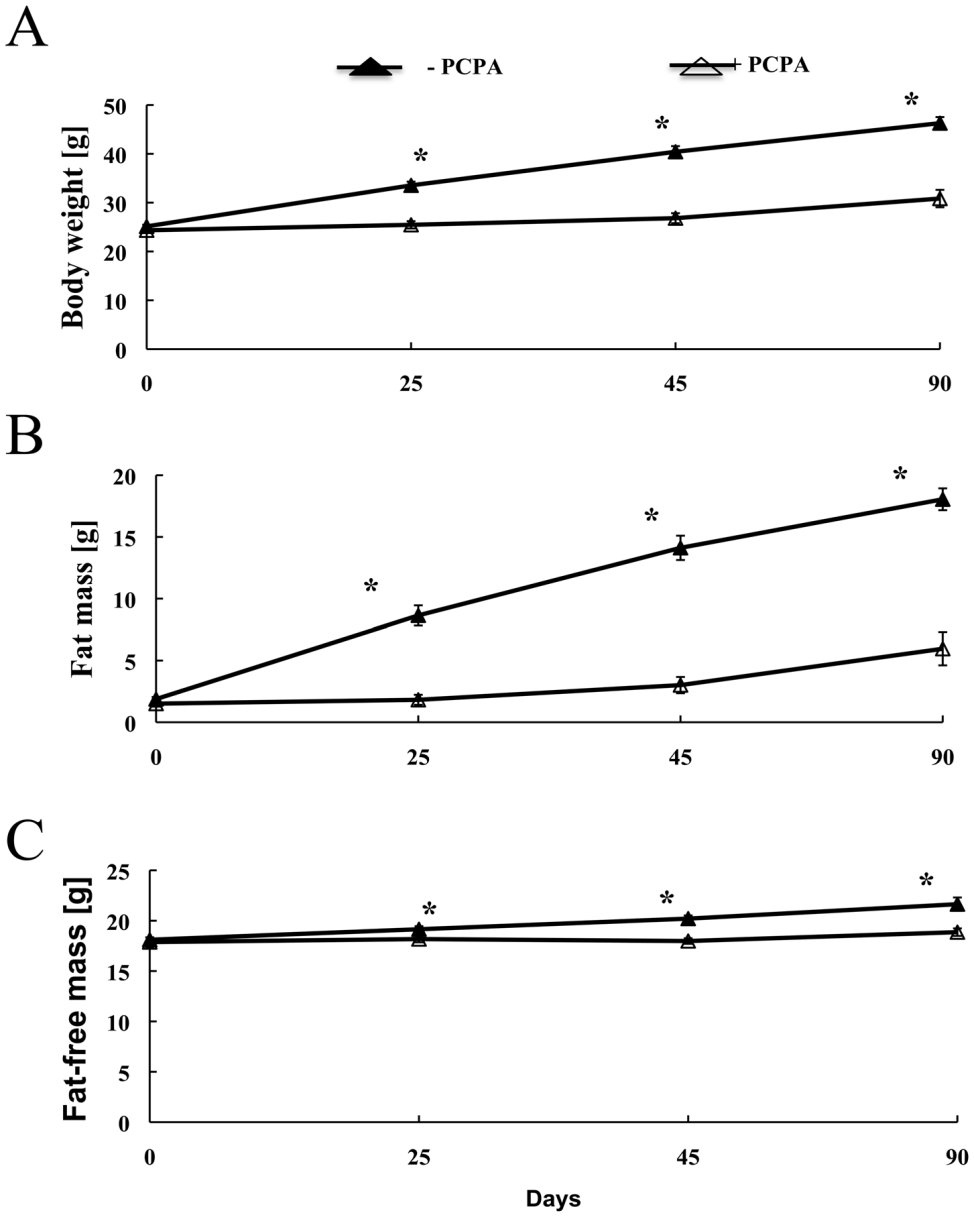
Mice treated with 2-PCPA for 90 days are resistant to obesity. Mice were fed a HFD with or without 2-PCPA for 90 days. Body weight measured with a scale (A), fat mass (B), and lean mass (C) measured using Magnetic Resonance Spectroscopy are shown. Lines represent means ± standard errors of each group (n = 5). (*) indicates a statistically significant difference (p<0.05) between the 2-PCPA-treated and non-treated groups.

**Figure 2:**
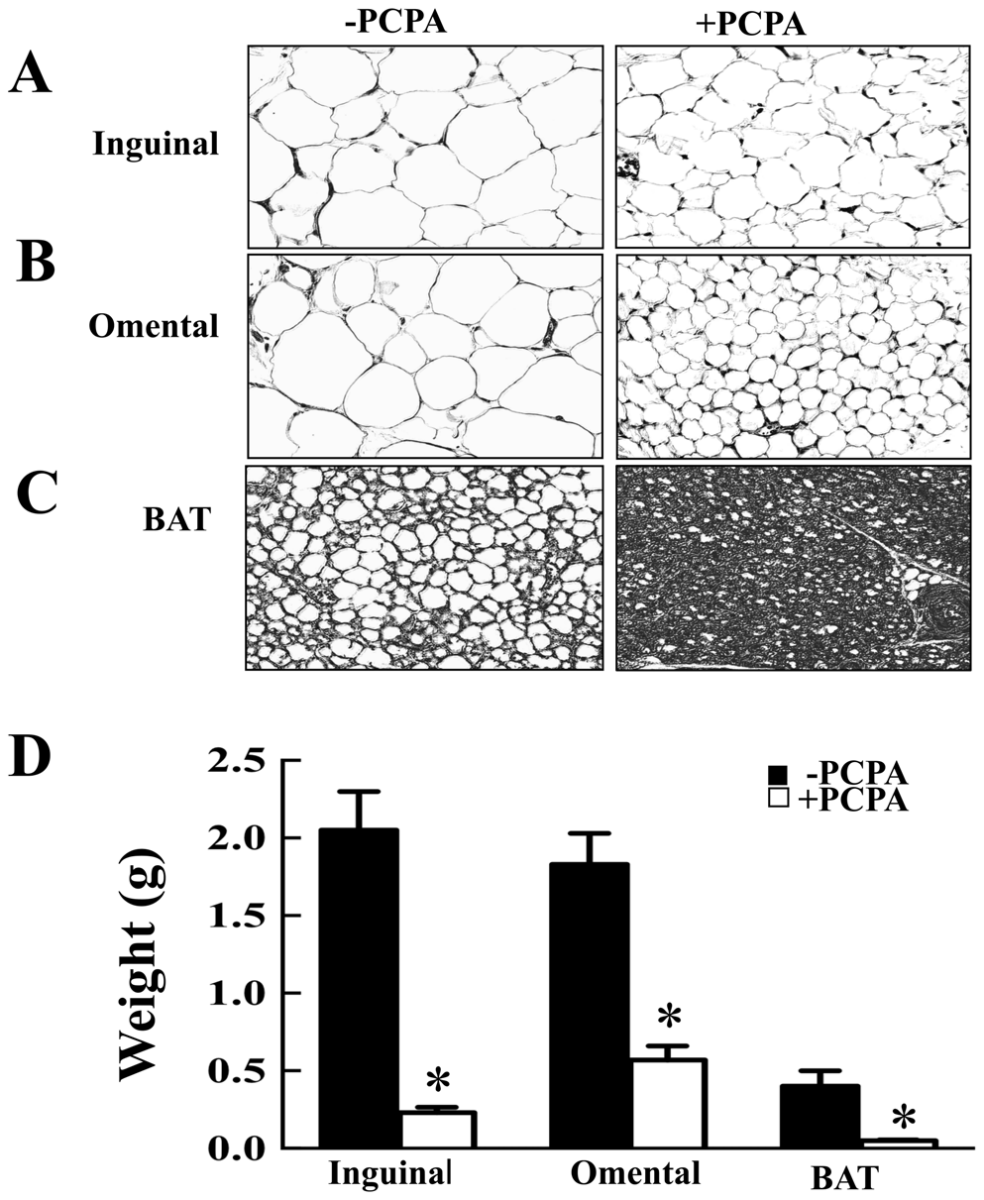
2-PCPA reduces triglyceride accumulation in white and brown adipose tissues. Mice fed a HFD with or without 2-PCPA for 90 days were sacrificed and adipose tissues were weighed and sectioned for morphologic assessment. (A, B, C) H&E staining of inguinal, omental, and brown adipose tissue sections, respectively. (D) Fresh tissue weights. Bars represent means ± standard errors of each group (n = 5). (*) indicates a statistically significant difference (p<0.05) between the 2-PCPA treated and non-treated groups.

2-PCPA treatment over 90 days resulted in a substantial protection against HFD-induced weight gain (Fig. 1A). The weight gain protection was primarily due to reduction in fat mass (Fig. 1B) with a relatively smaller change in lean mass (Fig. 1C). Histologically, adipocyte hypertrophy was reduced in inguinal (Fig. 2A) and omental (Fig. 2B) adipose tissues of 2-PCPA-treated mice. Although HFD is also known to increase neutral lipid accumulation in brown adipose tissue [5], 2-PCPA treatment reduced lipid accumulation in brown adipocytes (Fig. 2C). Consistent with these data, 2-PCPA decreased the dry weights of dissected inguinal and epididymal white adipose tissues as well as interscapular brown adipose tissue (Fig. 2D).

### 2-PCPA Protects Against HFD Induced Glucose Intolerance and Insulin Resistance

**Figure 3:**
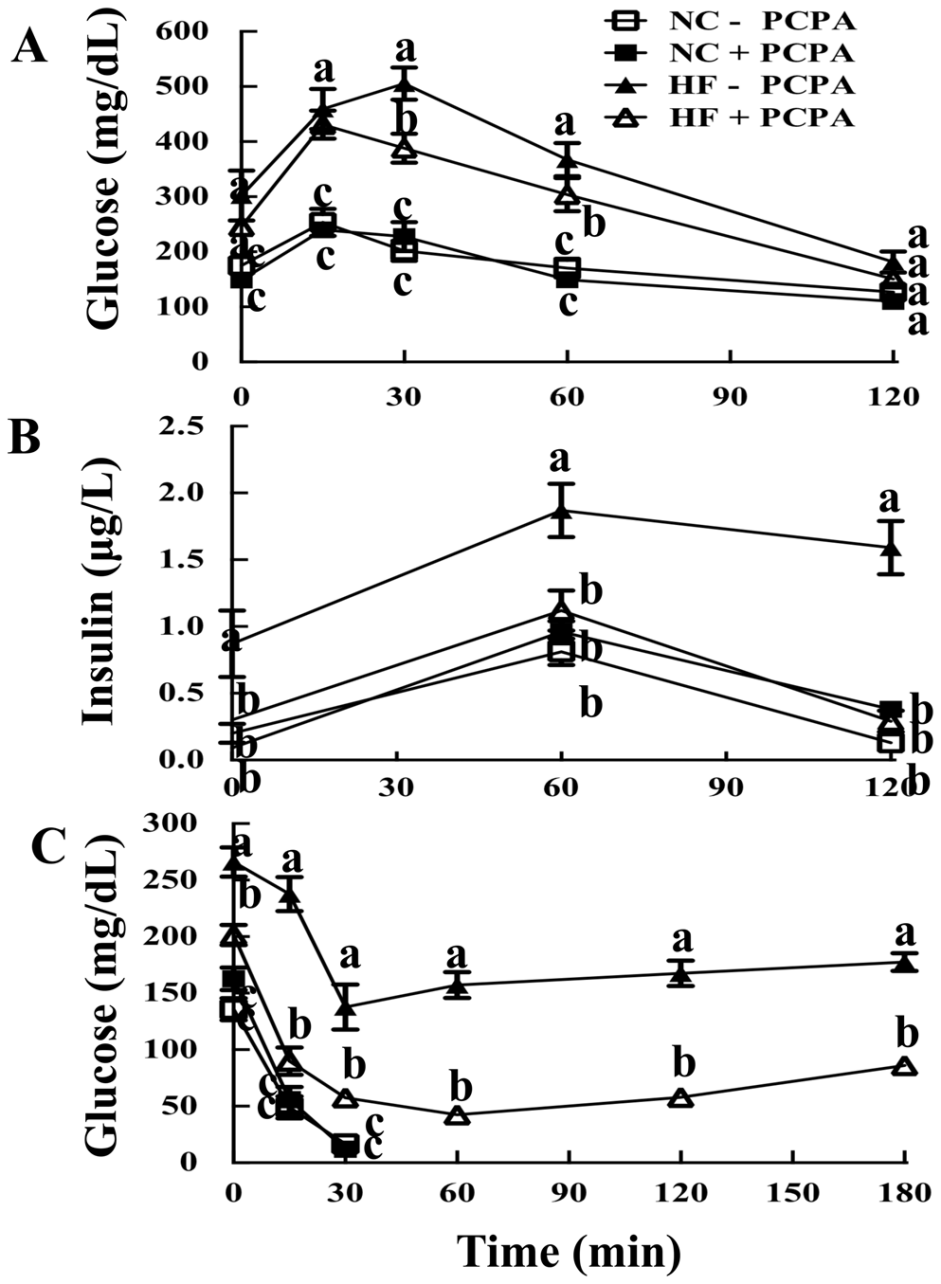
Mice treated with 2-PCPA are more glucose and insulin tolerant. Mice fed a HFD with or without 2-PCPA for 90 days were fasted for five hours and 1 g glucose/kg lean mass was injected intraperitoneally. Blood was drawn at different time points for measurements of plasma glucose (A) and insulin (B). (C) To determine tolerance to insulin, mice were fasted for four hours and injected intraperitoneally with 1 U insulin/kg lean mass. Glucose was measured at different time points. Lines represent means ± standard errors of each group (n = 5). Statistical analysis was performed using a two-way ANOVA followed by the Student-Newman-Keuls multiple range test. Identical letters indicate data points that are not statistically different from each other (p<0.05).

C57Bl6/J mice maintained on a NCD displayed an expected normal glucose tolerance when challenged with an intraperitoneal glucose load of 1 mg/kg lean mass (Fig. 3A). Essentially identical glucose tolerance was obtained for NCD fed mice whose food was supplemented with 2-PCPA for 90 days. As expected, mice fed a HFD for 90 days had both fasting hyperglycemia and a marked increase in the area under the curve indicating glucose intolerance (Fig. 3A). HFD-fed mice treated with 2-PCPA displayed an intermediate level of fasting hyperglycemia along with an improved glucose tolerance curve compared to mice on a HFD alone. In parallel, fasting insulin levels were higher in the HFD-fed mice and remained consistently elevated during the glucose tolerance test (Fig. 3B). In contrast, the insulin levels of NCD-fed mice and HFD-fed mice with 2-PCPA treatment were consistent with a normal insulin response to the glucose load. Moreover, HFD-fed mice were markedly insulin resistant whereas insulin tolerance was substantially improved in HFD-fed mice that also received 2-PCPA treatment (Fig. 3C). Overall, the improvement in insulin tolerance was greater than that observed in glucose tolerance tests suggesting that 2-PCPA probably protected against HFD-induced adipose and skeletal muscle insulin resistance but not liver insulin resistance.

### Long-term 2-PCPA Treatment Does not Increase Energy Expenditure but Reduces Food Intake

**Figure 4:**
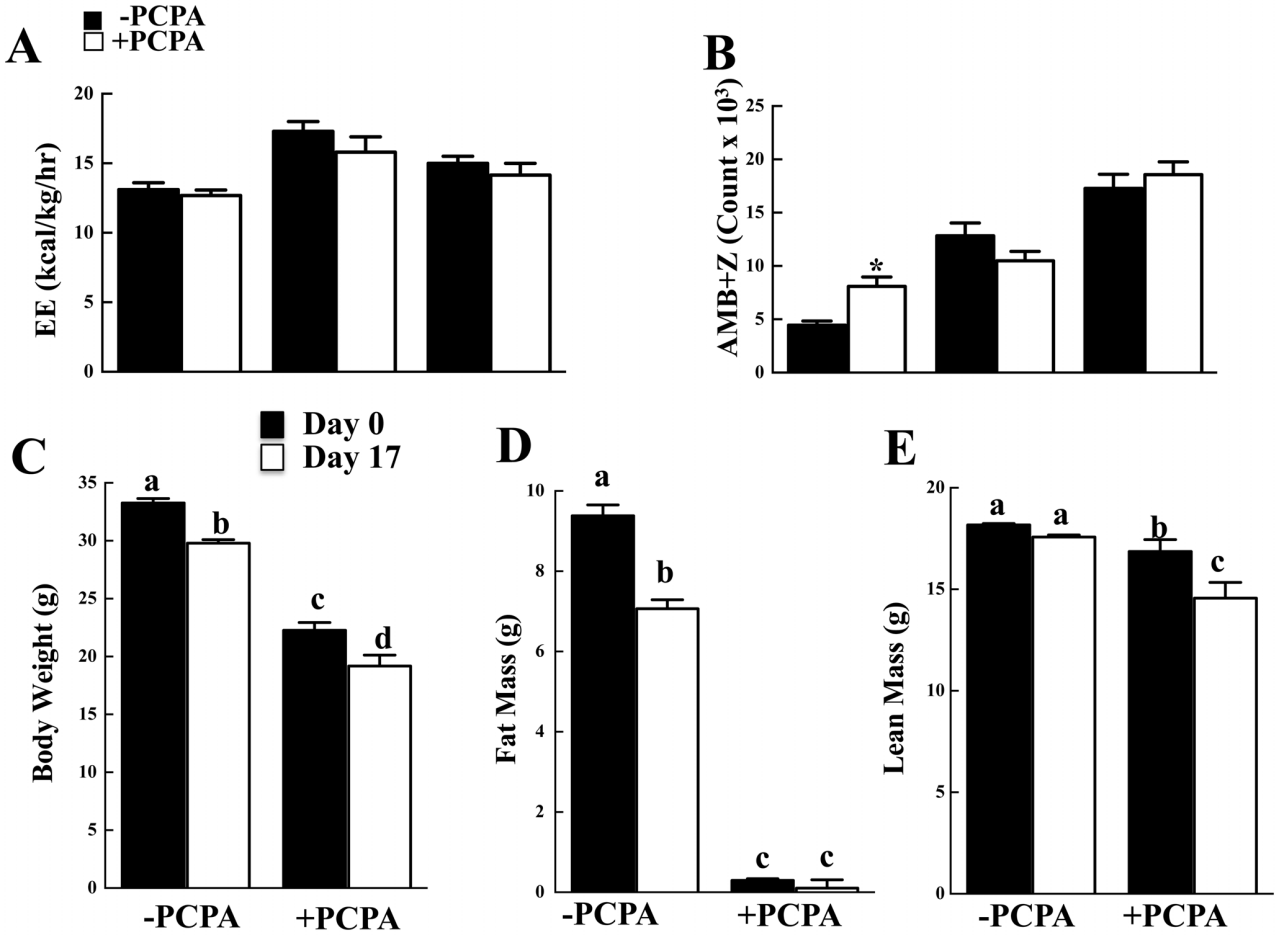
Mice treated with 2-PCPA for 90 days do not exhibit increased energy expenditure. Indirect calorimetry was carried out on mice fed a HFD with or without 2-PCPA for 90 days. (A) Energy expenditure. (B) Sum of ambulatory and vertical movements. (C) Daily food intake as measured every other day throughout the experiment. n = 5 in each group. The bars represent means ± standard errors. (*) indicates a statistically significant difference (p<0.05) between the 2-PCPA treated and non-treated groups. Mice were fed a HFD with or without 2-PCPA for 21 days and fed ad libitium. The mice were then restricted to 70% of their individual food intake for 17 days. (C) Body weight. (D) Fat mass. (E) Lean mass. n = 5 in each group. In panels (A) and (B) the bars represent means ± standard errors. (*) indicates a statistically significant difference (p<0.05) between the start (day 0) and end (day 17) of caloric restriction. In panels (C) and (D) statistical analysis was performed using a two-way ANOVA followed by the Student-Newman-Keuls multiple range test. Identical letters indicate data points that are not statistically different from each other (p<0.05).

To determine if the protection against HFD-induced obesity was due to increased energy expenditure, control and 2-PCPA treated mice were subjected to indirect calorimetry. 2-PCPA treatment had no significant effect on energy expenditure in either the light or dark cycles (Fig. 4A) even though there was a significant increase in spontaneous locomotor activity during the light (Fig. 4B). The increase in activity during the light cycle was accompanied by an apparent decrease, although not significantly different, in the dark cycle such that the total activity remained unchanged. We were also unable to detect any 2-PCPA-dependent increase in protein expression of UCP1 or the mitochondrial marker VDAC in brown adipose tissue (Figure S1A), inguinal (Figure S1B), or epididymal (Figure S1C) white adipose tissues. In addition, there was no morphological appearance of white adipose tissue browning (data not shown).

Examining weight loss that occurs during a short-term caloric restricted diet is an alternative way to assess energy expenditure. It is well established that mice placed on a caloric restricted diet display improved metabolic characteristics with the establishment of a stable lower energy balance [6]. As shown in Figure 4C, HFD-fed mice lost approximately 3.5 gm of total body mass 17 days after the initiation of 70% caloric restriction. This was predominantly accounted for by a loss of fat mass (2.3 gm) with a relatively small decline in lean mass (Fig. 4C,D). The HFD-fed mice treated with 2-PCPA had reduced total body mass and following caloric restriction lost a similar amount of body mass (3.1 gm) over the same time frame. However, due to the marked reduction in fat mass before caloric restriction, the 2-PCPA treated mice had no additional reduction in fat mass but lean mass was notably reduced (2.7 gm) (Fig. 4D,E). The differential loss of body mass (fat versus fat-free) following caloric restriction is consistent with the essential absence of fat mass in the 2-PCPA treated mice. Moreover, the weights of both groups of caloric restricted mice (with and without 2-PCPA treatment) eventually stabilized (data not shown). Thus, these data suggest that the weight reduction following 2-PCPA treatment was not due to enhanced basal energy expenditure.

**Figure 5:**
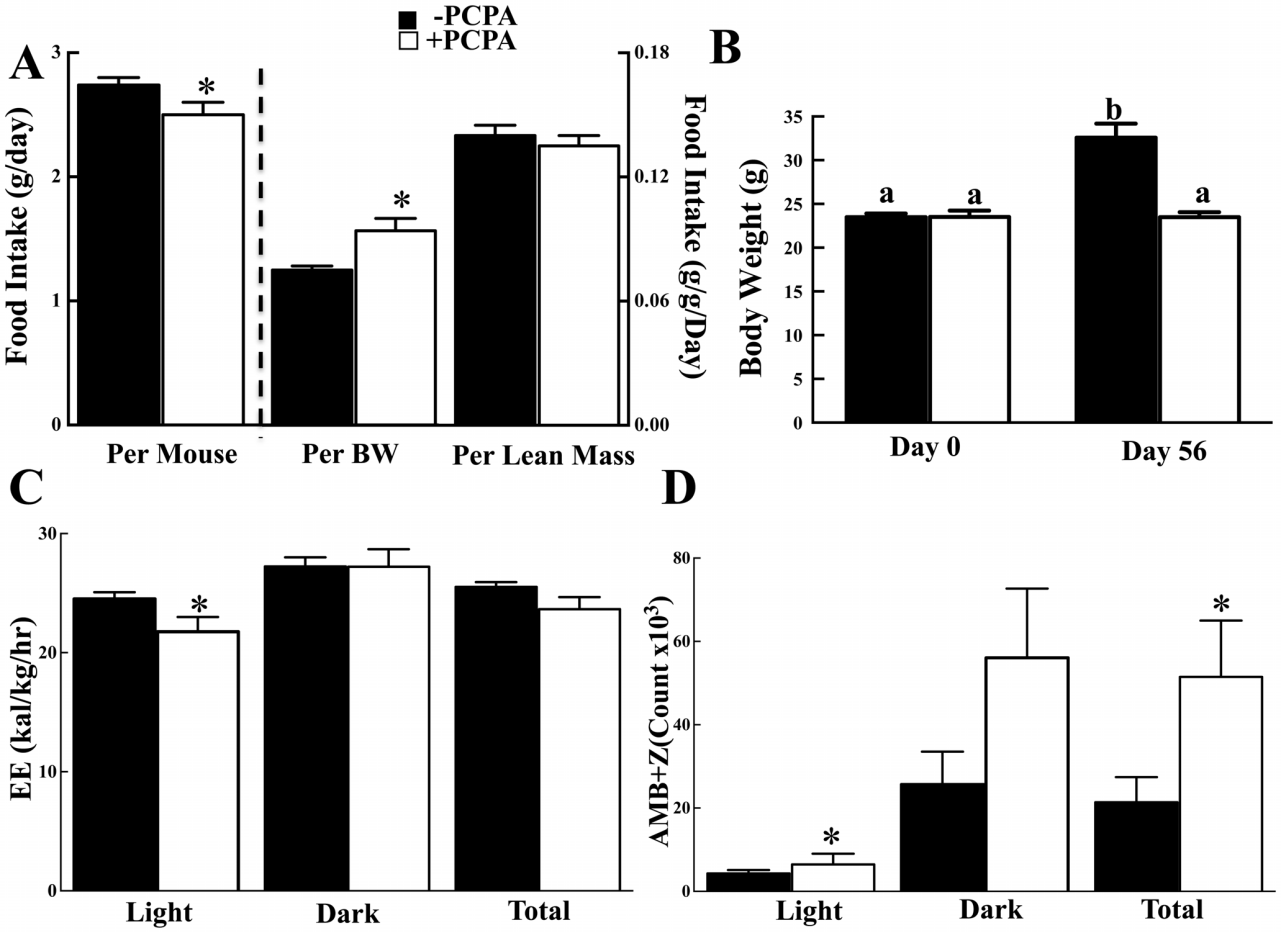
PCPA treatment has no effect on caloric intake but increases locomotor activity. Mice were fed HFD for 7 weeks and injected PCPA intraperitoneally every other day for a total of three times during the last week. (A) Food intake was normalized per mouse, per body weight and per lean mass. (B) Body weights and fat masses of HFD-fed mice were measured immediately prior to PCPA injection and two days after the third injection. Indirect Calorimetry was carried out immediately after the third injection. (C) Average energy expenditure during the 12-hour light cycle from 7 am to 7 pm and the 12-hour dark cycle from 7 pm to 7 am. (D) The sum of ambulatory and vertical movements. Mice were fed a HFD with or without 2-PCPA for 21 days and fed ad libitium. The mice were then restricted to 70% of their individual food intake for 17 days. (C) Body weight. (D) Fat mass. (E) Lean mass. n = 5 in each group. In panels (A) and (B) the bars represent means ± standard errors. (*) indicates a statistically significant difference (p<0.05) between the start (day 0) and end (day 17) of caloric restriction. In panels (C) and (D) statistical analysis was performed using a two-way ANOVA followed by the Student-Newman-Keuls multiple range test. Identical letters indicate data points that are not statistically different from each other (p<0.05).

Since 2-PCPA-treated mice were protected against HFD-induced obesity but did not spend more energy than control mice or display markedly increased activity, we next examined food intake. Long-term 2-PCPA treatment that was accomplished by mixing the drug in the food resulted in a reduction of approximately 0.24 g/day food intake (Fig. 5A). However, when food intake is normalized per total body weight the PCPA treated mice ate approximately 18 g/kg/day more whereas if normalized for lean mass there was no significant difference in food intake suggesting that differences in caloric intact did not account for the protection against obesity and that another mechanism(s) was responsible.

**Figure 6:**
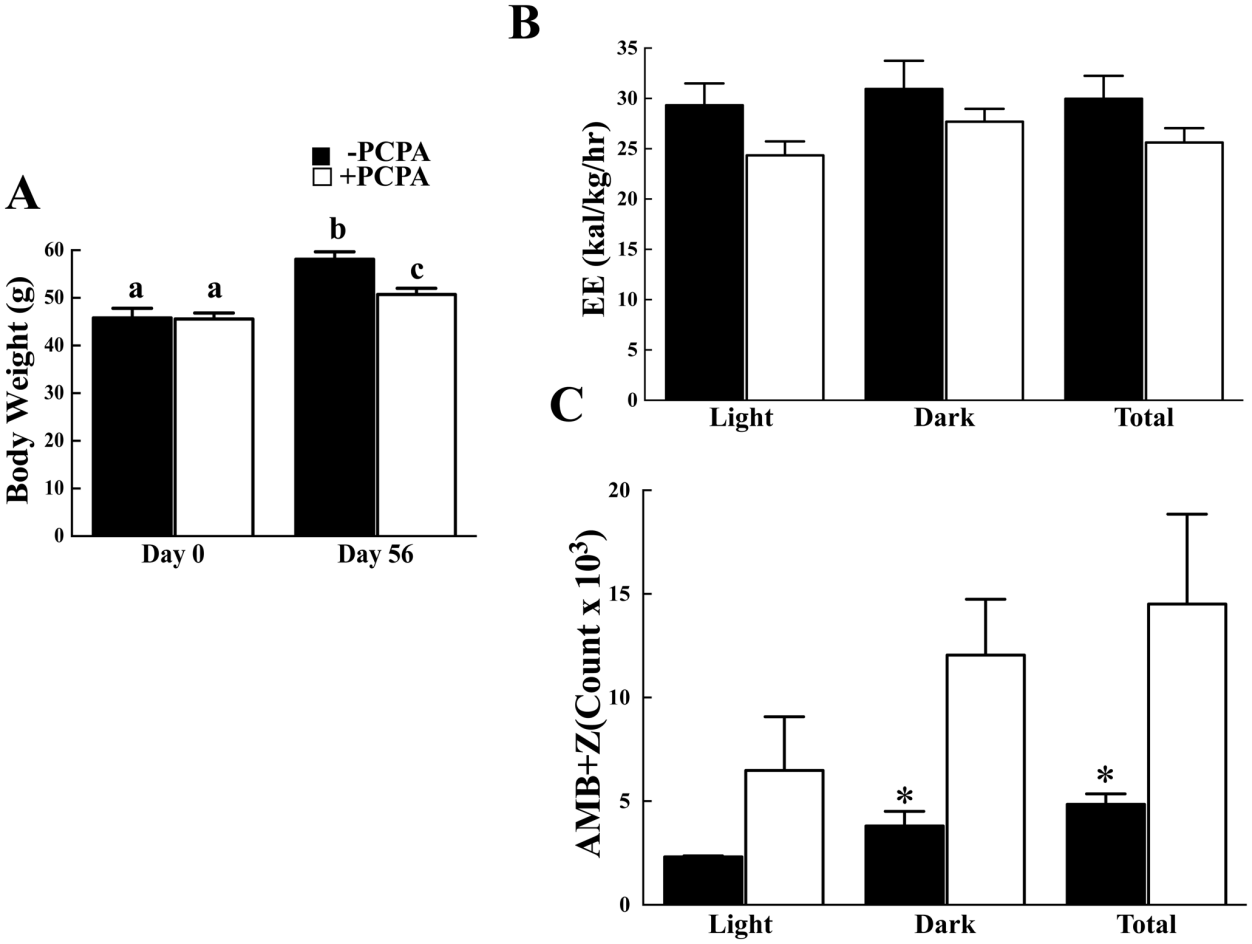
2-PCPA treatment reduces adiposity in ob/ob mice associated with increased locomotor activity. Pair-fed ob/ob mice were maintained on a normal chow diet for 7 weeks and then injected PCPA intraperitoneally every other day for a total of three times during the last week. (A) Body weights were initially measured and then two days after the third injection. Indirect Calorimetry was carried out immediately after the third injection. (B) Average energy expenditure during the 12-hour light cycle from 7 am to 7 pm and the 12-hour dark cycle from 7 pm to 7 am. (C) The sum of ambulatory and vertical movements. Panels (B) and (C) error bars represent means ± standard errors of each group (n = 5). (*) indicates a statistically significant difference (p<0.05) between the 2-PCPA treated and non-treated groups. In panel (A), statistical analysis was performed using a two-way ANOVA followed by the Student-Newman-Keuls multiple range test. Identical letters indicate data points that are not statistically different from each other (p<0.05).

To address this, we next pair-feed control and 2-PCPA treated mice on a high fat diet. Caloric intake for each set of mice was not significantly different (Fig. S2A) yet 2-PCPA was still able to induce a reduced body weight (Fig. 5B) due to decreased adipose tissue mass (Fig. S2B). This again occurred with no significant change in energy expenditure (Fig. 5C) but was associated with increased spontaneous locomotor activity particularly in the light cycle (Fig. 5D) and improved glucose tolerance (Fig. S2C). Similarly, we took advantage of ob/ob mice that are hyperphagic and display marked increases in fat mass. To eliminate any potential effect of 2-PCPA on food intake, the mice were again pair-fed to matched daily caloric intake (Fig. S3A). Under these conditions the 2-PCPA treated ob/ob mice gained less body weight than the control treated ob/ob mice (Fig. 6A), with no significant difference in energy expenditure (Fig. 6B). The reduced body weight resulted from decreased adipose tissue mass with improvement in glucose tolerance (Fig. S3B, S3C). However, there was again an increase in spontaneous locomotor activity that was more prevalent in the light cycle (Fig. 6C). Taken together, these data suggest that the ability of chronic 2-PCPA treatment to induce weight loss and decrease adiposity is not due to changes in energy expenditure but most likely results from an increase in activity.

### Short-term 2-PCPA Treatment Increases Activity and Energy Expenditure during the Light Cycle without Affecting Food Intake

**Figure 7:**
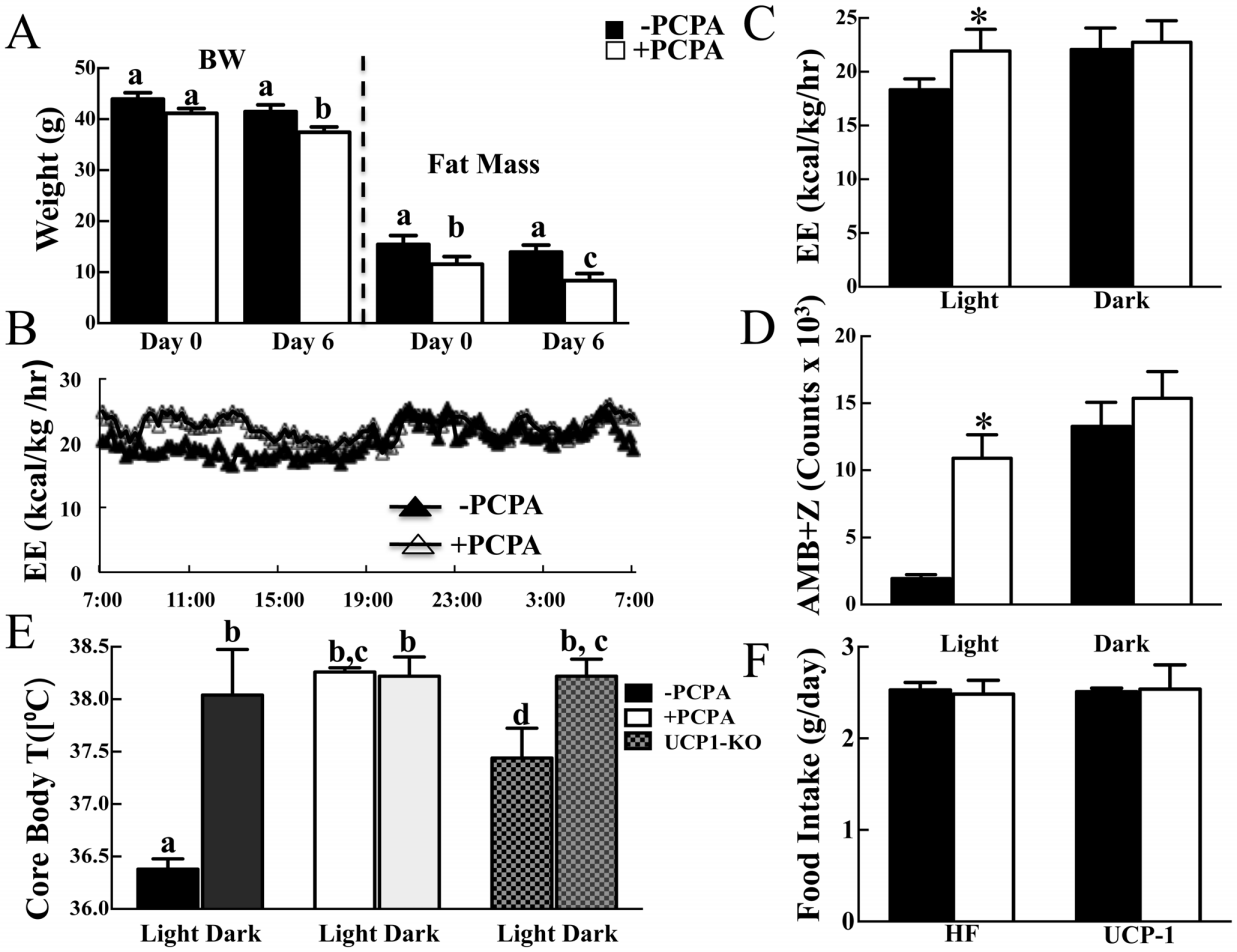
Short-term PCPA treatment increases locomotor activity and energy expenditure in the light cycle. Mice were fed HFD for 7 weeks and injected PCPA intraperitoneally every other day for a total of three times during the last week. (A) Body weights and fat masses of HFD-fed mice were measured immediately prior to PCPA injection and two days after the third injection. Indirect Calorimetry was carried out immediately after the third injection. (B, C) Average energy expenditure during the 12-hour light cycle from 7 am to 7 pm and the 12-hour dark cycle from 7 pm to 7 am. (D) The sum of ambulatory and vertical movements. (E) Core body temperatures of HFD-fed WT or UCP1-KO mice 24 hrs following the second PCPA injection. The measurement was taken in the light cycle unless otherwise noted. (F) Daily HFD intake during the six days of short-term experiment. Bars represent means ± standard errors of each group (n = 5). (*) indicates a statistically significant difference (p<0.05) between PCPA treated and non-treated groups unless otherwise noted.

It has been previously reported that acute 2-PCPA treatment results in transcriptional activation of gene markers for increased energy expenditure and elevated mitochondrial respiration, so it was concluded that 2-PCPA increases energy expenditure [4]. Since the apparent long-term protection against HFD-induced obesity was not due to increased energy expenditure, we examined the effect of acute intraperitoneal injections of 2-PCPA (Fig. 7). In this case, mice were initially placed on a HFD for seven weeks and then injected every other day with 2-PCPA for six days while maintained on the HFD.

As observed in the long-term treated mice, intraperitoneal injections of 2-PCPA over six days resulted in reduced body weight that was accounted for by a decrease in fat mass (Fig. 7A). However, unlike long-term 2-PCPA treatment, short-term treatment did not affect food intake (Fig. 7F) and increased energy expenditure during the light but not the dark cycle (Fig. 7B,C). In this case, the increased energy expenditure correlated with a substantial increase in spontaneous locomoter activity in the light but not dark cycle (Fig. 7D).

During the light cycle, when mice are resting, the core body temperature of HFD-fed mice was approximately 36.4°C (Fig. 7E). In the dark cycle, when mice are active, core body temperature increased to approximately 38°C. Short-term 2-PCPA treatment increased core body temperature in the light cycle also to 38°C that was essentially the same as core body temperature in the dark cycle. The 2-PCPA induced increased core body temperature in light cycle is consistent with the increased spontaneous locomotor activity suggesting that the mice remain more active in the light cycle.

### Short-term 2-PCPA Treatment Reduces Adiposity in HFD-induced Obese UCP1 Null Mice

**Figure 8:**
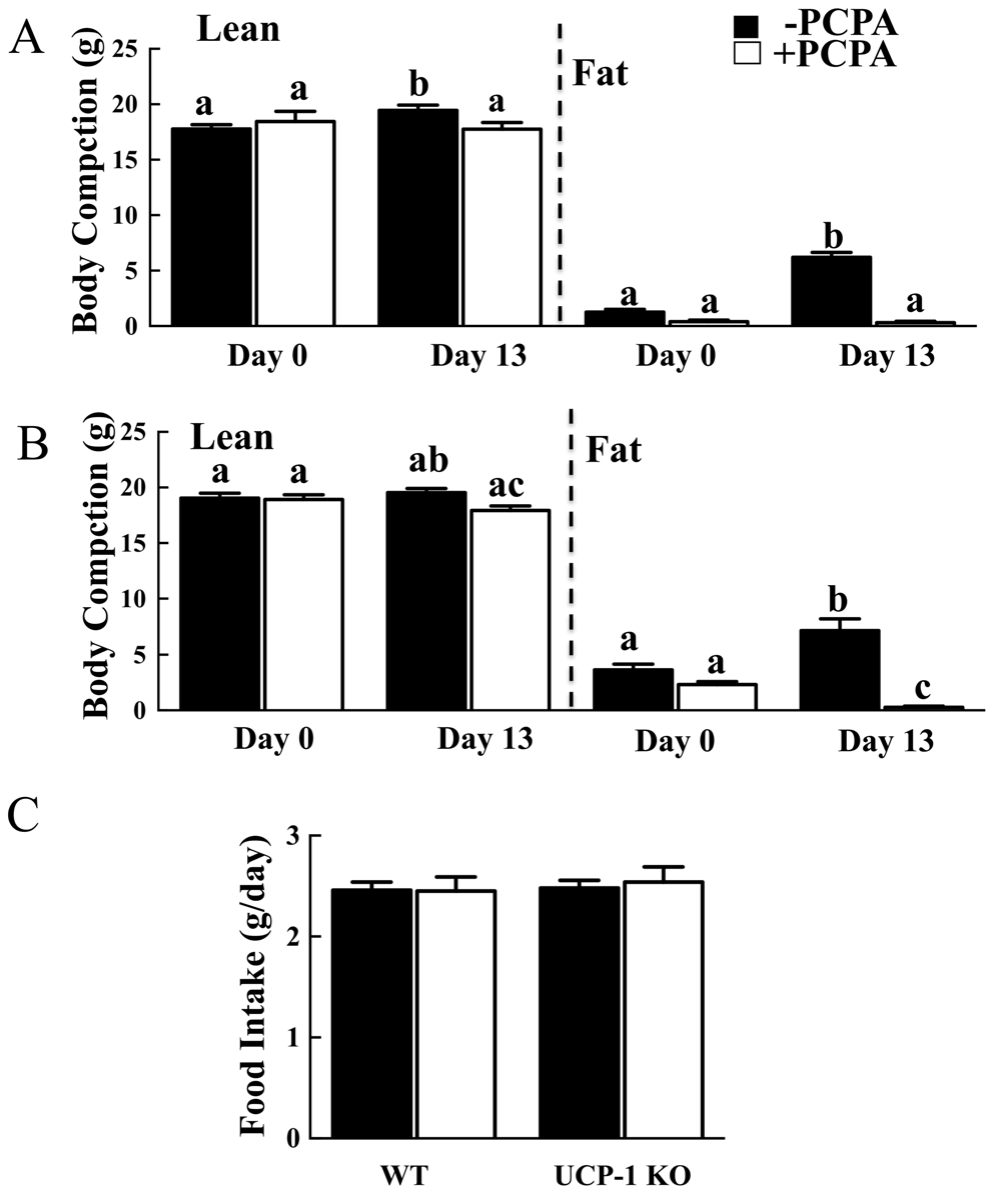
PCPA reduces adiposity of obese UCP1-KO mice. WT or UCP1-KO mice were fed a HFD with or without PCPA for 13 days. Fat and lean masses of WT (A) and UCP1-KO (B) mice prior to (day 0) and following (day 13) HFD feeding. (C) Daily food intakes of WT and UCP1-KO mice. In panels (A) and (B), bars represent means ± standard errors of each group (n = 5). (*) indicates a statistically significant difference (p<0.05) between PCPA treated and non-treated groups. In panel (C), statistical analysis was performed using a one-way ANOVA followed by the Student-Newman-Keuls multiple range test. Identical letters indicate data points that are not statistically different from each other (p<0.05).

It has been previously suggested that 2-PCPA can increase sympathetic tone to brown adipose tissue through its inhibition of monoamine oxidase, and increased sympathetic tone induces expression of UCP1 [7]. Although we were unable to detect changes in adipose tissue UCP1 protein expression, we further assessed whether 2-PCPA can enhance thermogenic energy expenditure by examining the effect of genetic UCP1-deficiency. Consistent with our previous findings, WT mice fed a HFD for 13 days gained approximately 5 grams fat mass whereas 2-PCPA treated mice displayed no significant increase in fat mass (Fig. 8A). Similarly, UCP1-KO mice gained about 3.5 grams fat mass whereas 2-PCPA-treated mice lost about 2 grams (Fig. 8B). Neither strain lost lean mass due to 2-PCPA treatment and there was no statistically significant difference in food intake (Fig. 8C). In addition, short-term 2-PCPA treatment increased core body temperature of UCP1-KO mice in the light cycle (Fig. 7E) consistent with an increase in activity as observed for the wild type mice (Fig. 7D). These data demonstrate that the ability of 2-PCPA to reduce adiposity occurred through a UCP1-independent mechanism.

## Discussion

Obesity is reaching epidemic proportions in the United States with approximately 35% of adults and 17% of children having a body mass index (BMI) greater than 30 and is the single strongest predictor of insulin resistance and the development of type 2 diabetes. Thus, identifying mechanistic and physiologic processes that reduce obesity and sequelae will have a significant therapeutic potential.

Monoamine oxidases catalyze the deamination of neurotransmitter amines and 2-PCPA inhibition of this activity has been clinically used to treat depression [2]. In addition to its antidepressant effect, 2-PCPA has been reported to cause anorexia in rodents. This effect was dose-dependent and correlated with elevated brain neurotransmitter amines [8]. However, recent findings implicate 2-PCPA in elevating basal energy expenditure through its peripheral inhibition of adipose tissue lysine-specific demethylase 1 (LSD1), resulting in increased expression of genes that are related to increased energy expenditure. To show that 2-PCPA affected metabolism in-vivo, 2-PCPA was injected intraperitoneally and resistance to obesity without affecting food intake was demonstrated [4]. However, whether 2-PCPA-induced increase in gene expression or oxygen consumption in insulin-stimulated 3T3-L1 adipocytes translated to a physiologic increase in energy expenditure remains unresolved.

Based upon these data, we examined the in-vivo physiologic properties of 2-PCPA treatment in HFD-fed mice. Our feeding regimen followed that described in a report of an anti-obesity effect in long-term-fed mice that was not due to reduced food intake [1]. Even though our 2-PCPA-treated mice exhibited resistance to obesity and improved insulin resistance, energy expenditure was unchanged whereas food intake was reduced compared to control mice based upon total intake but was not different when normalized for lean mass and was actually more when normalized for total body mass, suggesting that energy consumption was not a contributing factor to the anti-obesity effect of 2-PCPA. Of note, both aforementioned studies reported a body mass difference of 4–5 grams between 2-PCPA treated and control groups at the end of the experiment. In our study, long-term treatment lasted longer and the difference in body mass was approximately 16 grams. It is therefore possible that the reduced food intake in our experiment may be secondary to adaptation to reduced body weight. Consistent with this conclusion, the effectiveness of 2-PCPA was also observed in pair-feed ob/ob mice supporting a food intake independent effect on 2-PCPA.

Thus, to further examine if 2-PCPA treatment could bring about resistance to obesity by affecting energy expenditure, we had to bypass its effect on food intake. To this end, we adopted a short-term 2-PCPA treatment approach. Although we observed increased energy expenditure, it was only in the light cycle. This directly correlated with a substantial increase in spontaneous locomotor activity, which is in agreement with one of the drug’s side effects, arousal [9], [10]. Core body temperature was also elevated only in the light cycle to levels usually observed during the normal active dark cycle. The ability of 2-PCPA to increase body temperature in the light cycle was UCP1 independent, and 2-PCPA-treated UCP1-KO mice were similarly resistant to obesity as WT mice. Consistent with these findings, 2-PCPA treatment of pair-fed wild type high fat diet fed mice or pari-fed normal chow feed ob/ob mice also displayed increased spontaneous locomotor activity.

Thus, in both the acute and long-term, oral administration of 2-PCPA the only metabolic parameter that was associated with reduced weight gain was increased locomotor activity. Thus, we conclude that in contrast to previous studies, the ability of 2-PCPA to decrease adiposity occurs independent of increases in basal energy expenditure and food intake and reflects alterations in physical activity.

## Supporting Information

Figure S1 **Long-term PCPA treatment does not induce UCP1 and VDAC expression in white and brown adipose tissue.** Western blots depicting UCP1 and VDAC expression in BAT (A), inguinal (B), and epididymal (C) adipose tissues of mice fed a HFD with or without PCPA for 90 days. Each lane represents a single tissue lysate. BAT in white adipose tissue blots is used as a positive control since it has relatively high expression levels of UCP1 and VDAC. GAPDH serves as a loading control.
(TIF)

Figure S2 **PCPA treatment decreases fat mass and improves glucose tolerance independent of food intake in wild type mice.** Wild type C57Bl6/J mice were placed on a HFD pair-fed to match total daily caloric intake for 6 weeks with and without 2-PCPA. (A) Daily food intake. (B) Lean and fat mass. (C) Glucose tolerance at the end of the 42 day feeding period. In panels A and C, data represent means ± standard errors of each group (n = 5). (*) indicates a statistically significant difference (p<0.05) between PCPA treated and non-treated groups unless otherwise noted. In panel (B), statistical analysis was performed using a one-way ANOVA followed by the Student-Newman-Keuls multiple range test. Identical letters indicate data points that are not statistically different from each other (p<0.05).
(TIF)

Figure S3 **PCPA treatment decreases fat mass and improves glucose tolerance independent of food intake in the ob/ob genetic obese and insulin resistant mice.** The genetic obese ob/ob mice were placed on a normal chow diet and pair-fed to match total daily caloric intake for 6 weeks with and without 2-PCPA. (A) Daily food intake. (B) Lean and fat mass. (C) Glucose tolerance at the end of the 42 day feeding period. In panels A and C, data represent means ± standard errors of each group (n = 5). (*) indicates a statistically significant difference (p<0.05) between PCPA treated and non-treated groups unless otherwise noted. In panel (B), statistical analysis was performed using a one-way ANOVA followed by the Student-Newman-Keuls multiple range test. Identical letters indicate data points that are not statistically different from each other (p<0.05).
(TIF)
