# Supplementary figures and images for: The Antidepressant Trans-2-Phenylcyclopropylamine Protects Mice from High-Fat-Diet-Induced Obesity
Source: PLoS One. 2014 Feb 21;9(2):e89199. doi: 10.1371/journal.pone.0089199 (PMC3931726; doi:10.1371/journal.pone.0089199)

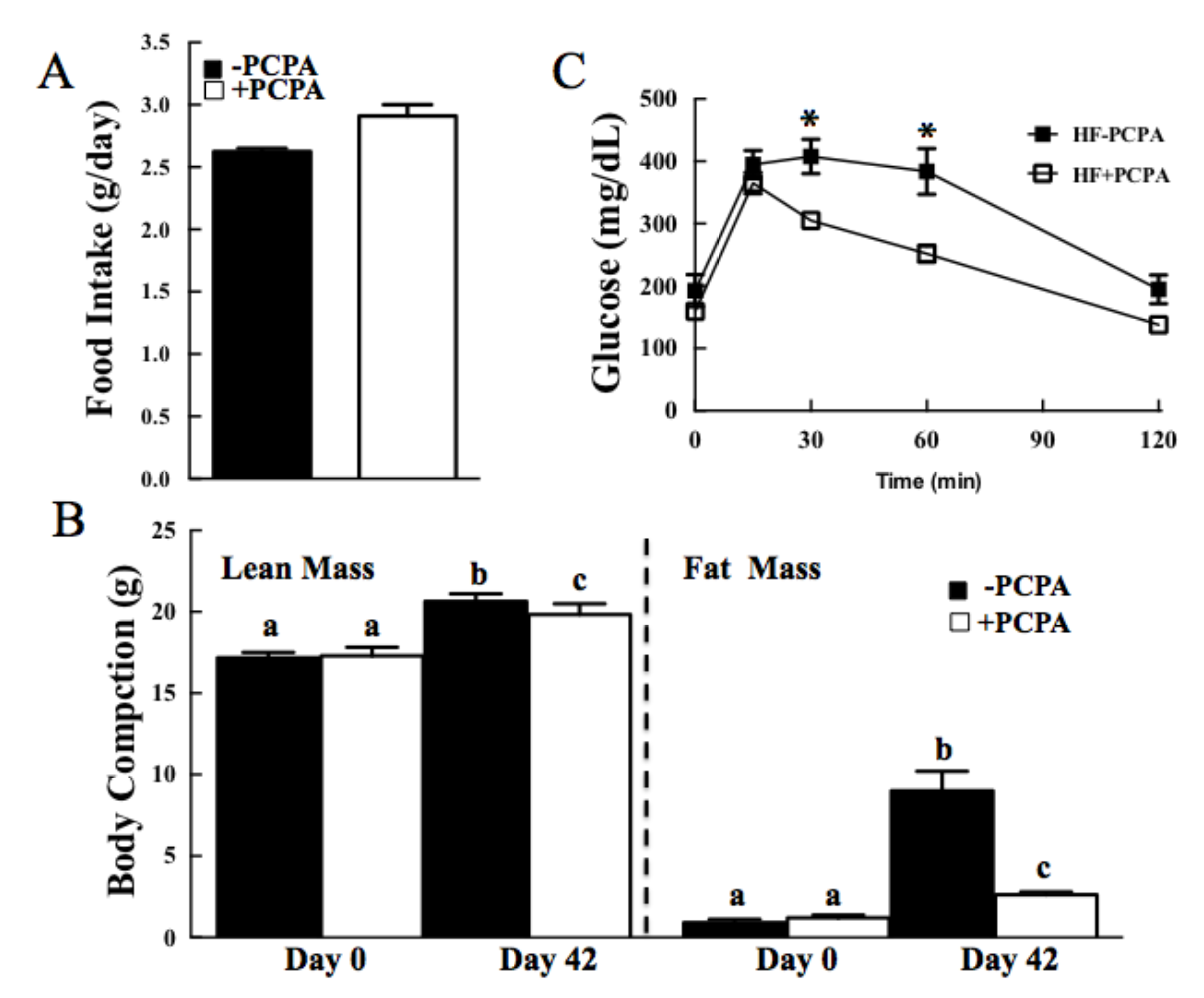

Supplement: Figure S2 — PCPA treatment decreases fat mass and improves glucose tolerance independent of food intake in wild type mice. Wild type C57Bl6/J mice were placed on a HFD pair-fed to match total daily caloric intake for 6 weeks with and without 2-PCPA. (A) Daily food intake. (B) Lean and fat mass. (C) Glucose tolerance at the end of the 42 day feeding period. In panels A and C, data represent means ± standard errors of each group (n = 5). (*) indicates a statistically significant difference (p<0.05) between PCPA treated and non-treated groups unless otherwise noted. In panel (B), statistical analysis was performed using a one-way ANOVA followed by the Student-Newman-Keuls multiple range test. Identical letters indicate data points that are not statistically different from each other (p<0.05). (TIF) [file pone.0089199.s002.tif]
